# Editorial: Trait mining and genetic enhancement of millets and potential crops: modern prospects for ancient grains

**DOI:** 10.3389/fpls.2023.1291893

**Published:** 2023-09-28

**Authors:** Dinesh C. Joshi, Salej Sood, Himabindu Kudapa, Meiliang Zhou, Dipak Santra

**Affiliations:** ^1^ Division of Crop Improvement, Vivekananda Institute of Hill Agriculture, Indian Council of Agricultural Research (ICAR), Almora, Uttarakhand, India; ^2^ Division of Crop Improvement, Central Potato Research Institute, Indian Council of Agricultural Research (ICAR), Shimla, Himachal Pradesh, India; ^3^ Center of Excellence in Genomics and Systems Biology, International Crops Research Institute for the Semi-Arid Tropics, Hyderabad, India; ^4^ Institute of Crop Sciences, Chinese Academy of Agricultural Sciences, Beijing, China; ^5^ Department of Agronomy and Horticulture, University of Nebraska, Lincoln, NE, United States

**Keywords:** millets, potential crops, nutritional security, climate resilience, genomics

Over the last decades, agricultural productivity has witnessed a significant increase. Yet, only 12 crops provide 75% of the world’s food supplies and three major crops, rice, wheat and maize, provide 50% of global dietary requirements (Joshi et al., 2019). These leading cereals have inherently low micronutrient concentrations, and overreliance on these crops leads to micronutrient deficiencies (Joshi et al., 2020). Sub-Saharan Africa and South Asian countries are the hot spots where the prevalence of micronutrient malnutrition is very high (Harding et al., 2018). For instance, In India alone, over 80% of the population is at risk of calcium deficiency and up to 25% suffer from iron and zinc deficiency (Ritchie et al., 2018). Many low-volume, high-value crops cultivated for millennia have high nutritional quality and can contribute to global food security and help combat hidden hunger. Therefore, dietary diversification by including micronutrients and vitamins rich minor and neglected food crops in the existing cropping systems is one of the most effective ways of sustainably reducing hidden hunger (Joshi et al., 2018). Millets and pseudocereals have the ability to provide a reasonable yield in the harshest environmental conditions of the world. Their ability to withstand environmental stresses and fragile ecosystems makes them ideal dual-purpose crops for grain and fodder production in low-input marginal agricultural systems prevalent in semi-arid regions. However, millets and pseudocereals have received little scientific attention and the control of economic traits remains unknown (Joshi et al., 2019; Sood et al., 2019).

This Research Topic accentuated the nutritional importance, breeding methods and genomic resources of different millets and pseudocereals. Millets, the richest source of micronutrients and gluten-free protein, are represented by pearl millet, little millet, proso millet and finger millet. Similarly, pseudocereals, which are the reservoir of high-quality protein and essential amino acids, are represented by amaranth, buckwheat and quinoa.

Most minor millets and pseudocereals are characterized by small florets and cumbersome floral biology. Therefore, realizing genetic gains through recombination breeding is still a daunting challenge. The review by Nagaraja et al. provides a holistic overview and state-of-the-art artificial hybridization techniques in minor millets. The success rate in generating true hybrids using various methods has been reported in different minor millets. It also describes a newly developed modified crossing method known as the Small Millets University of Agricultural Sciences Bengaluru (SMUASB) method in proso and little millets with high success rate of ~60% true hybrids.

Owing to their orphan status and small research community, genomic resources are poorly documented in minor millets. To address this issue, Francis et al. reported a total of 84 and 171 SNP markers in high and low-yielding mutants of proso millet, respectively. A total of three functional SNPs were correlated with the genes encoding ubiquitin-protein ligase. Similarly, Shekhar et al. developed a freely available comprehensive transcriptome database of little millet ((LMTdb) for the first time. The unique database comprised transcriptome sequence, functional annotation, microsatellite markers, differentially expressed genes and pathway information. These emerging genomic resources in minor millets provide a platform to the breeders for mining genes for developing nutritionally rich and climate-resilient cultivars.

Blast caused by *Magnaporthe* spp one of the most critical biotic constraints affecting many millet species, including pearl millet, finger millet, foxtail millet and barnyard millet. It affects the plant at all the growth stages, including leaf, neck and panicle stages and has been reported to cause yield loss of 70-80% in endemic and hot spot areas (Sood et al., 2023). However, limited information about the blast pathogen’s different virulent genes and host-plant interaction is available. Palanna et al. investigated ten virulent genes isolated from two hundred blast-infected samples from different millet crops grown in major hot spot locations in India. Of the ten genes, MPS1 and Mlc amplified in most tested isolates and can be used to develop blast-resistant cultivars.

Compared to minor millets, genomic resources in pseudocereals (amaranth, buckwheat and quinoa) are well documented. This is mainly because of the availability of well-assembled reference genomes in pseudocereals. To further enrich the genomic resources in buckwheat, Fang et al. identified 394 DEGs and 24 candidate genes regulating large grain size. Similarly, Xue et al. enriched the information about the second largest basic helix-loop-helix (bHLH) transcription factor superfamily and identified a total of 218 CqbHLH transcription factor genes located on 18 chromosomes of quinoa (*Chenopodium quinoa*). By virtue of its C_4_ photosynthetic pathway, grain amaranth is expected to be more tolerant to heat stress. To explore this aspect, Goel et al. conducted a genome-wide analysis and identified and characterized 13 heat shock factors imparting heat tolerance to *A. hypochondriacus* in the vegetative and reproductive stages. These enriched genomic resources in pseudocereals are expected to accelerate genetic gain through molecular breeding efforts in pseudocereals.

Of the total global pearl millet production, 45% comes from drylands, particularly in western and central Africa (Meena et al., 2021). Innovative agronomic practices are required to further boost the pearl millet production in drylands’ low input cultivation practices. Pilloni et al. confirmed through field and lysimeter trials that higher sowing density significantly increased the yield and water use efficiency of pearl millet genotypes. These results provide concrete evidence that pearl millet productivity can be enhanced by increasing the sowing density, especially in areas facing high evaporative demand.

Finally, papers in this Research Topic provide information on diverse aspects of millets and potential crops. Advances on these orphan crops have been made using scanty resources and international collaborations, but dedicated researchers and institutions have made respectable progress. This Research Topic does not cover the complete spectrum of millets and potential crops, and hopefully, this series can be continued, and the latest findings on finger millet, barnyard millets, foxtail millet, kodo millet, chia, ricebean, etc. can be included.

## Author contributions

DJ: Conceptualization, Writing – original draft, Writing – review & editing. SS: Writing – review & editing. HK: Writing – review & editing. MZ: Conceptualization, Writing – review & editing. DS: Conceptualization, Writing – review & editing.
